# Impact of controlled-release urea on rice yield, nitrogen use efficiency and soil fertility in a single rice cropping system

**DOI:** 10.1038/s41598-020-67110-6

**Published:** 2020-06-26

**Authors:** Zhaoming Chen, Qiang Wang, Junwei Ma, Ping Zou, Lina Jiang

**Affiliations:** Institute of Environmental Resources and Soil Fertilizer, Zhejiang Academy of Agricultural Sciences, Hangzhou, 310021 China

**Keywords:** Plant sciences, Agroecology

## Abstract

Overuse of nitrogen (N) fertilizer has led to low N use efficiency (NUE) and high N loss in single rice cropping systems in southeast China. Application of controlled-release urea (CRU) is considered as an effective N fertilizer practice for improving crop yields and NUE. Here, field experiments were conducted during 2015–2017 to assess the effects of two CRUs (resin-coated urea (RCU) and polyurethane-coated urea (PCU)) on rice yields, NUE and soil fertility at two sites (Lincheng town (LC) and Xintang town (XT)). Four treatments were established at each site: (1) control with no N application (CK), (2) split application of conventional urea (U, 270 kg N ha^−1^), (3) single basal application of RCU (RCU, 216 kg N ha^−1^), and (4) single basal application of PCU (PCU, 216 kg N ha^−1^). The N application rate in the CRU treatment compared to the U treatment was reduced by 20%. However, the results showed that, compared to split application of urea, single basal application of CRU led to similar rice grain yields and aboveground biomass at both sites. No significant difference in the N uptake by rice plant was observed between the U and CRU treatments at either site. There were no significant differences in the N apparent recovery efficiency (NARE) among the U, RCU and PCU treatments, with the exception of that in XT in 2015. Compared to application of U, application of CRU increased the N agronomic efficiency (NAE) and N partial factor productivity (NPFP) by 17.4–52.6% and 23.4–29.8% at the LC site, and 15.0–84.1% and 23.2–33.4% at the XT site, respectively, during 2015–2017. Yield component analysis revealed that greater rice grain yield in response to N fertilizer was attributed mainly to the number of panicles per m^2^, which increased in the fertilized treatments compared to the CK treatment. The application of CRU did not affect the soil fertility after rice harvest in 2016. Overall, these results suggest that single basal application of CRU constitutes a promising alternative N management practice for reducing N application rates, time- and labor-consuming in rice production in southeast China.

## Introduction

Rice (*Oryza sativa* L.) is one of the most important food crops in China and plays a vital role in guaranteeing national food security^1–3^. China is the largest rice producer worldwide and produces nearly 30% of the rice produced globally^4,5^. In 2016, 207 million tons of rice were produced in China, accounting for more than 30% of total food production^6^. Nitrogen (N) fertilizer plays an indispensable role in improving rice yield and quality^7^. More than 4 Tg per year of N fertilizer was applied for rice production in China from 2001 to 2010^8^. However, increases in crop yields are not linearly correlated with increases in the application rate of N fertilizer, which inevitably results in deceased the N use efficiency (NUE) and increased N losses^9,10^. Excessive use of N fertilizer has resulted in a series of environmental issues, such as surface water eutrophication, groundwater pollution, greenhouse gas emission and soil acidification^11–14^. Therefore, efficient N management is a crucial approach for increasing the NUE while minimizing environmental pollution in rice agro-ecosystems.

Various approaches to N management, such as multiple split application and deep placement, can improve both rice yields and NUE and reduce N losses^10,15–17^. However, these practices require more labor and knowledge of N management than do conventional practices or are limited by technology lag. Application of controlled-release urea (CRU) constitutes an effective practice for increasing crop yields and NUE while reducing N loss via ammonia volatilization (AV), nitrous oxide (N_2_O) production, surface runoff and leaching^12,18–20^. Numerous studies have shown that the application of CRU significantly increased rice grain yield and NUE compared to the application of traditional N fertilizer^7,21–23^. The results of a field experiment conducted by Li *et al*.^20^ showed that CRU significantly increased the grain yield of late rice and the apparent N recovery by 6–18% and 3–17%, respectively, compared to urea application at the same N rate. A review by Chalk *et al*.^18^ showed that the recovery of fertilizer N was higher, and the unaccounted N loss lower in response to a ^15^N-labeled CRU treatment than in response to a ^15^N-labeled urea treatment. In addition, several studies have reported that CRU could significantly reduce N_2_O, nitric oxide (NO) and methane (CH_4_) emissions from paddy soils compared to conventional urea^20,24,25^. Resin-coated urea (RCU) and polyurethane-coated urea (PCU) are two kinds of CRU that can increase both crop yields and NUE and reduce N loss^7,20,23,26,27^.

With respect to rice production, split application of N fertilizer is usually recommended for improving NUE and crop yields^28^. Compared to single application of urea, split application can significantly increase rice grain yields and NUE, but other studies have reported no significant difference in rice grain yield between a one-time application of urea and a split application of urea in central China^20^. Split application of N is more time consuming and labor intensive than is a single basal application^28,29^. In China, many rice farmers have part-time jobs in cities, which leads to limited time and labor for agriculture^30,31^. Furthermore, it is difficult for farmers to master the proper time of application and amount of topdressing of N fertilizer^32^. CRU is helpful for decreasing the use of N fertilizer and saving time and labor inputs^20,29^. Many studies have shown that CRU can be applied once as basal fertilizer with no effect on rice grain yields^7^.

NUE can be sub-classified in terms of N apparent recovery efficiency (NARE), N agronomic efficiency (NAE) and N partial factor productivity (NPFP)^33^. The NARE is used to describe the uptake efficiency of N fertilizer. The grain yield increase per unit N fertilizer applied is expressed by the NAE, and the NPFP represents the use efficiency of soil N and fertilizer N. Dobermann^34^ proposed that the recommended NARE, NAE and NPFP for good management are 50–80%, 25–30 kg kg^−1^ and 60–80 kg kg^−1^, respectively. The average values of NARE, NAE and NPFP of rice in China are 39.3%, 12.6 kg kg^−1^ and 48.6 kg kg^−1^, respectively^1^. Many studies have shown that NUE is much lower in China than in developed countries^35–37^.

The middle and lower Yangtze River (MLYR) basin is one of the most important agricultural regions in China^38^. Rice–wheat rotation, rice–rapeseed rotation and early rice–late rice rotation compose the main cropping systems in the middle and lower reaches of the Yangtze River^38^. In this region, the application rate of N fertilizer ranges from 200 to 300 kg ha^−1^, whereas the rice grain yield is only 6.7–7.6 t ha^−1^ ^39^. A review by Che *et al*.^1^ demonstrated that the NARE, NAE and NPFP of rice were 35–44%, 10–15 kg kg^−1^ and 29–68 kg kg^−1^ in the MLYR basin, respectively.

Thus, the objective of the present study was to compare the effects of CRU and U on rice yield, NUE and soil fertility from 2015 to 2017 in the MLYR basin, China. We hypothesized that application of CRU would increase the grain yield and NUE of rice and would not affect soil fertility.

## Results

### Weather conditions

**Figure 1:**
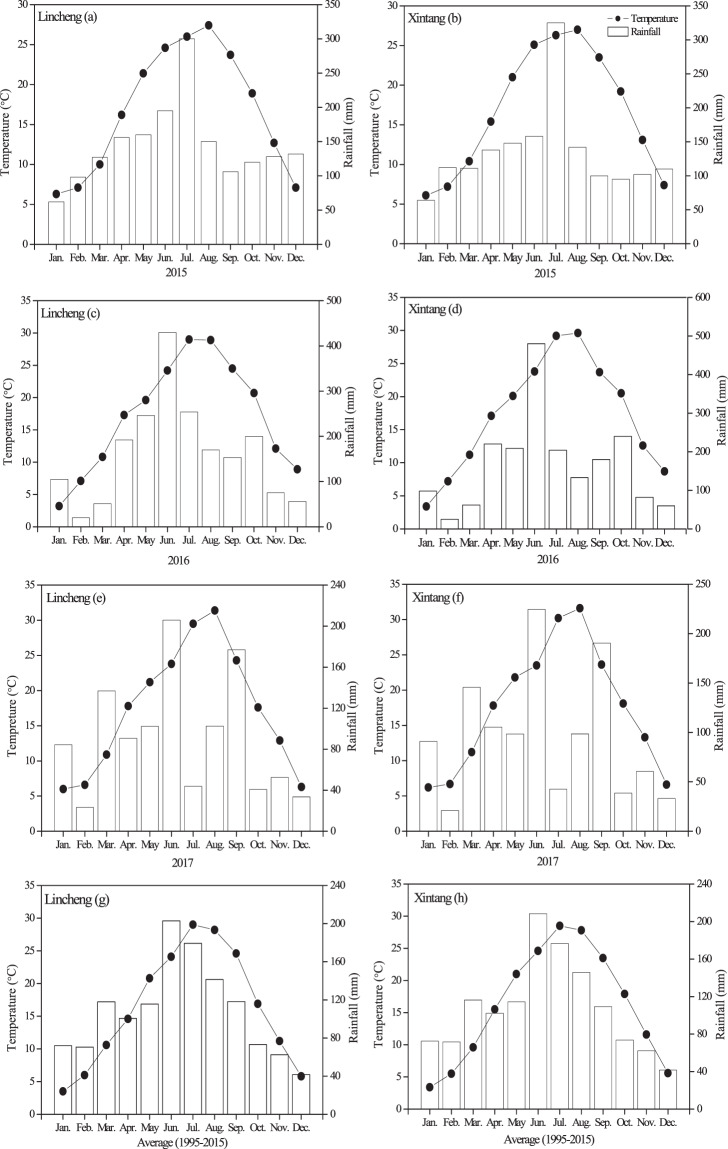
Monthly mean rainfall and temperature at the two experimental sites during the 2015 to 2017 rice growing season.

The monthly temperature and rainfall were recorded from rice transplantation to harvest during the 2015 to 2017 rice growing seasons at the two sites (Fig. 1). The average temperatures were 24.2 (19.2–26.3 °C), 25.5 °C (20.7–28.9 °C) and 25.3 °C (17.6–31.4 °C) in LC and 24.1 (18.9–27.4 °C), 25.4 °C (20.5–29.6 °C) and 25.4 °C (18.1–31.6 °C) in XT during the 2015 to 2017 rice growing seasons, respectively. The total rainfall during the rice growing season was 820, 1207 and 570 mm at the LC site, and 871, 1237 and 595 mm at the XT site, in 2015, 2016 and 2017, respectively (Fig. 1). The rainfall data during the rice growing season was 710 mm in long-term averages at both sites, accounting for 55% of total rainfall of whole year, respectively. The rainfall distributions were different among the three years at both sites. The total rainfall during the rice growing season was greater in 2016 than in 2015 and 2017 at both sites.

### Grain yield and aboveground biomass

**Figure 2:**
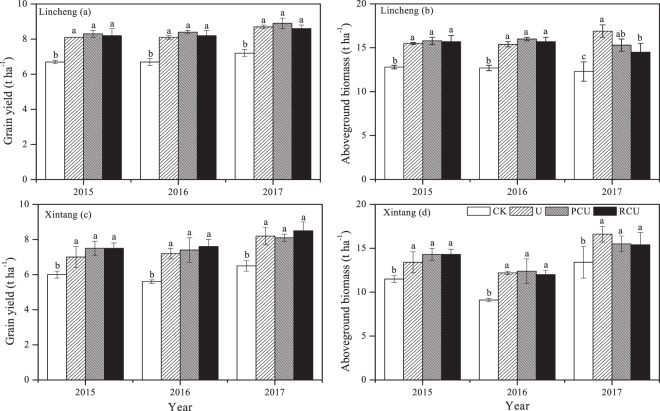
The grain yield and aboveground biomass of rice were affected by the different treatments at the two experimental sites during the 2015 to 2017 rice growing season. The values are presented as the mean ± standard deviation (n = 3). The different letters represent significant differences at the level of *P* < 0.05. CK, U, RCU and PCU represent no N application, conventional urea, resin-coated urea and polyurethane-coated urea, respectively.

The rice grain yield was significantly affected by treatment (T) but was not influenced by year (Y) or their interaction at both sites (Table S1). The rice grain yield in the CK treatment was 6.7–7.2 t ha^−1^ in LC and ranged from 5.6 to 6.5 t ha^−1^ in XT from 2015 to 2017 (Fig. 2a,c). The grain yield of rice in the N fertilizer treatments (U, RCU and PCU) significantly increased by 21.1–23.1%, 21.2–25.8% and 19.6–23.3% at the LC site and by 16.5–24.3%, 28.4–35.6% and 24.2–30.3% at the XT site compared to those in CK in 2015, 2016 and 2017, respectively. The N rate in the CRU treatments was reduced by 20% relative to that in the U treatment from the 2015 to 2017 rice season (Table 1). However, no significant differences in rice grain yield was observed between the U and CRU treatments at either site (Fig. 2a,c). Similarly, applications of RCU and PCU led to similar grain yields at both sites. Moreover, single CRU application reduced the N fertilization time and reduced the work force needed for rice production relative to the spilt application of urea in the paddy fields.

**Table 1:** Application rate and timing of fertilizers for the four treatments at the two experimental sites during the 2015 to 2017 rice growing seasons.

Site	Treatment	Basal fertilizer N: P_2_O_5_: K_2_O (kg ha^−1^)	Tillering fertilizer N: P_2_O_5_: K_2_O (kg ha^−1^)	Booting fertilizer N: P_2_O_5_: K_2_O (kg ha^−1^)
Lincheng	CK	0: 80: 120		
	U	135: 80: 120	54: 0: 0	81: 0: 0
	RCU	216: 80: 120		
	PCU	216: 80: 120		
Xintang	CK	0: 80: 120		
	U	135: 80: 120	54: 0: 0	81: 0: 0
	RCU	216: 80: 120		
	PCU	216: 80: 120		

CK, U, RCU and PCU represent no N application, conventional urea, resin-coated urea and polyurethane-coated urea, respectively.

Treatment significantly affected the aboveground biomass of rice in LC, and year and treatment significantly influenced the aboveground biomass in XT (Table S1). However, the interactions of Y × T had no impacts on the aboveground rice biomass at both sites. The aboveground biomass of rice in the CK treatment was 12.7–12.8 t ha^−1^ in LC and 9.1–13.4 t ha^−1^ in XT (Fig. 2b,d). The combined results for the three years and two sites showed that N fertilizer treatments (U, RCU and PCU) resulted in significantly increased aboveground biomass of rice compared to the CK treatment. When the different N fertilizers were compared, the results concerning the aboveground biomass were very similar to those concerning the grain yield, and no significant difference was found between the U and CRU treatments (Fig. 2b,d), except for that at the LC site in 2017. In addition, there was no significant difference between the RCU and PCU at either site.

### N concentration and N uptake

The N concentration in grain was not influenced by year, treatment and their interaction in LC, but in XT, year significantly affected the N concentration in the grain (Table S1). Year and treatment had significant effect on the N concentration in straw in LC, and only treatment had significant effect on the N concentration in straw in XT. In 2015, no significant differences in the rice grain N concentration were observed among the treatments, but compared to that in the CK treatment, the straw N concentration in the N fertilizer treatments significantly increased at the LC site (Table 3). The N concentration in the grain was higher in the CRU treatments than in the CK treatment, but there was no significant difference between the CRU and U treatments at the XT site in 2015. The N fertilizer treatments presented greater straw N concentrations than did the CK treatment. The N concentrations in the grain and straw were significantly higher in the N treatments than in the CK treatment in both LC and XT in 2016, with the exception of the PCU treatment in XT. There were no differences in the N concentrations in rice among the U, RCU and PCU treatments at the LC or XT site (Table 3). In 2017 rice season, the N concentration in grain was significantly higher in the N fertilizer treatments than in the CK treatment in LC. The highest N concentration in straw was observed in the U treatment, and there was no significant difference among the CK, RCU and PCU treatments in LC. The N concentration in grain ranged from 11.9 to 13.3 mg kg^−1^ in XT, and there was similar among the treatments. The U treatment had a higher N concentration in straw than the CK and PCU treatments.

N uptake by the rice grain was significantly affected by treatment but was not influenced by treatment and the Y × T interaction in LC (Table S1). While N uptake by the grain was both significantly affected by year and treatment. Both year and treatment significantly influenced N uptake by the rice straw and N uptake by the whole rice plant. In 2015, the N fertilizer treatments significantly increased N uptake by the grain, straw and whole plant compared to CK in both LC and XT (Table 3). There were no differences in N uptake by rice plants between the U and CRU treatments in both LC and XT. The N uptake by the grain, straw and rice plants were significantly increased by the application of N fertilizer at both sites in 2016 (Table 3). The RCU and PCU treatments presented similar levels of N uptake by the grain, straw and whole plant as the U treatment at the LC and XT site in 2016. In 2017, the N uptake by the grain, straw and whole plant were significantly increased by the N fertilizer treatments, compared to CK in both LC and XT. No significant differences were observed in the N uptake by grain and whole rice plant among the U, RCU and PCU treatments in both sites. The highest N uptake by straw was recorded in the U treatment in both sites, and the lowest value was found in the CK treatment.

### N use efficiency

Year, treatment and the interaction of Y × T had no significant effect on the NARE at both sites (Table S1). Only the treatment significantly affected the rice NAE at either site. The NPFP was only significantly affected by the treatment at both sites. However, year and the Y × T interaction were both no significant. In the 2015 rice growing season, the NARE, NAE and NPFP ranged from 21.6 to 24.5%, 5.3 to 7.2 kg kg^−1^ and 30.1 to 38.3 kg kg^−1^ at the LC site, respectively (Table 4). All the NUE indices (NARE, NAE and NPFP) were significantly higher in the CRU treatments (RCU and PCU) than in the U treatment at the XT site in 2015. In LC in 2016, the NARE in the CRU treatments was similar to that in the U treatment (Table 4). The NARE was 22.6%, 27.1% and 21.3% in the U, RCU and PCU treatments in XT in 2016, respectively. The CRU treatment increased the NAE and NPFP by 39.4–56.8% and 28.2–32.0%, respectively, compared to the U treatment. In 2017, there were no significant differences in NARE among the treatments at both sites. PCU application significantly increased the NAE compared to the U treatment in XT, while no significant difference was found among the three treatments in LC. The NPFP was significantly higher in the CRU treatments (RCU and PCU) than in the U treatment in both sites.

### Components of grain yield

Year and treatment significantly affected the panicles per m^2^ in LC, while treatment and Y × T interaction had significant in XT (Table S1). In LC, only year significantly influenced the grains per panicle, grain filling percentage and 1000-grain weight but the treatment and the Y × T interaction had no effects. The effects of year on the grains per panicle and grain filling percentage were significant in XT. 1000-grain weight was not significantly affected by treatment in XT. CK consistently showed the lowest panicle numbers (Table 5). The applications of U, RCU and PCU significantly increased the number of panicles per m^2^ by 5.6–45.5%, 14.0–41.1% and 14.0–24.7%, respectively (Table 5). In 2015, the number of grains per panicle was significantly lower in the CK treatment than in the N fertilizer treatments at the LC site. However, the lowest number of grains per panicle was recorded in the RCU treatment at the XT site. In 2016, no significant differences were observed in the number of grains per panicle among the treatments at either site. In 2017, the RCU treatment had the highest number of grains per panicle in LC. In XT, the grains per panicle was 116–126 in the N treatments, and was significant higher than in the CK treatment. Moreover, there were no differences in the grain filling percentage among the four treatments in LC from 2015–2016. But in 2017, the grain filling percentage was significantly lower in the U treatment than in other treatments. In XT, the grain filling percentage was lower in the U treatment than in the PCU treatment in 2015 but was not significantly different among the treatments in 2016. The highest grain filling was found in the U treatment in 2017. With the exception of that in LC in 2015, the 1000-grain weight did not significantly differ among the treatments.

### Soil chemical properties

After the rice harvest in 2016, there were no significant differences in soil pH among the treatments in LC; however, the CRU treatments significantly reduced the pH levels by 8.0–8.2% compared to the CK treatment in XT (Table 6). The pH was similar between the CRU treatments and the U treatment. No significant differences in SOM were found among the treatments at either site. The soil TN was significantly lower in the CRU treatments (RCU and PCU) than in the CK treatment. However, with respect to soil TN, there were no significant differences among the U, RCU and PCU treatments. The Soil AHN ranged from 202 to 218 mg kg^−1^ in LC and 196 to 239 mg kg^−1^ in XT. Soil available P was 6.22–7.75 mg kg^−1^ and 14.24–17.64 mg kg^−1^ in LC and XT, respectively (Table 6). No significant differences were observed in soil available K among the four treatments at the three sites in 2016.

## Discussion

The average rice grain yield was 8.0 t ha^−1^ at the LC site and 7.3 t ha^−1^ at the XT site. These are within the range of results (5.9–9.0 t ha^−1^) from the middle and lower reaches of the Yangtze River^1,39^. The average grain yield in response to the CK was 6.5 t ha^−1^ in this study (Fig. 1a, c), indicating a large supply of soil indigenous N. The N rate in the CRU treatment was reduced by 20% relative to that in the U treatment from the 2015 to 2017 rice growing season (Table 1). However, the results from the present study showed no significant differences in rice grain yield between the U treatment and CRU treatments at either site from 2015 to 2017 (Fig. 2). These results reveal that a single basal application of CRU can maintain rice grain yields even if the N application rate is reduced by 20% compared to that in the split application of urea. Furthermore, a single basal application of CRU can reduce the amount of topdressing applied and save labor and time. Similarly, Li *et al*.^20^ reported that a single basal application of CRU did not significantly affect the grain yield of late rice compared to a split application of urea in Central China. Furthermore, a 2-year field experiment conducted by Ke *et al*.^32^ revealed that the rice grain yield was significantly lower in a sulfur-coated urea and polymer-coated urea treatment than in a U treatment in the Taihu region of China. However, other studies have showed that CRU produced a higher rice grain yield than did U at the same rate^22,26,40^. These differences in results may be caused by different environmental conditions, soil types and rice varieties in these studies. Grant *et al*.^29^ reported that the benefits of CRU were markedly influenced by the environmental conditions of the region, such as temperature and soil moisture.

The NARE is an important index that is used to express to fertilizer N uptake efficiency^41^. A single basal application of CRU was shown to increase the NARE by 23–48% compared to a split application of traditional urea during rice production^7,22,41,42^. N is slowly released from CRU and is available for plant uptake, which results in a reduction in N loss and thereby increases the NUE of rice^20^. Many studies have suggested that CRU treatments can strongly reduce ammonia volatilization from paddy field compared to the U treatment^19,20,40^. In the present study, the NARE increased with decreasing N application rates as follows: 22.0% and 24.9% in the U and CRU treatments in LC and 18.7% and 21.4% in the U and CRU treatments in XT, respectively (Table 4). Similarly, Xu *et al*.^17^ reported that the NARE of rice increased from 29.6% to 42.6% when the N rate decreased from 300 kg ha^−1^ to 180 kg ha^−1^ in southeastern China. The NAE is another index and measures the response of yield to increases in the N rate^41^. The recommended NAE for good management is 25–30 kg kg^−1^1^37^. In our study, the NAE was 5.2–8.0 kg kg^−1^ at the LC site and 3.7–9.4 kg kg^−1^ at the XT site (Table 4), both of which were much lower than the recommended values. The main explanation is that the grain yield was lower and the N application rate was higher in our study than in other studies^37^. Some researchers recommend 180 kg N ha^−1^ for rice production in the middle and lower reaches of the Yangtze River^37^. In the current study, the NPFP ranged from 29.9 to 41.1 kg kg^−1^ at the LC site and from 26.0 to 39.4 kg kg^−1^ at the XT site (Table 4). A two-year field experiment conducted by Liu *et al*.^37^ showed that the NPFP of rice was 38.2–67.8 kg kg^−1^ in the middle and lower reaches of Yangtze River, which was higher than the result of our study. The main reason for this difference is that the rice grain yield was lower in our study than in the previous study. Our results showed that the CRU treatment had a higher NPFP than did the U treatment. Reducing the N application rate will improve the NPFP^37^. However, Miao *et al*.^41^ found that single applications of CRU reduced the NPFP compared to the split application of urea in bowl-seedling machine-transplanted rice. Moreover, the N release rate of CRU is greatly affected by the temperature and soil moisture^29,41^.

Many studies have shown that application of N fertilizer can improve soil chemical properties in agricultural ecosystems^43,44^. Xue *et al*.^45^ found that N fertilized treatments increased the SOM and TN after 3-years experiment in a paddy soil. However, the results of our study showed that compared to the CK, the application of N fertilizer did not affect the SOM and TN concentrations (Table 6). The reason maybe that the initial soil chemical properties, such as the SOM, TN and AHN, were relatively high in our study (Table 2). Therefore, short-term N application does not affect the soil fertility in the paddy soils in this region. Previous studies in China have indicated that the soil acidification could be caused after three-year application of N fertilizer in a paddy soil in southeast China^46^. Similarly, the soil pH decreased over the two-year N fertilizer treatments in XT. In the present study, there were no significant differences in AHN concentration between the CRU treatments and U treatment (Table 6). In contrast to our results, Zheng *et al*.^44^ reported that two-year application of CRU could increase the available N concentration within the 0–20 cm soil layer compared to conventional N fertilizer. A possible reason is that the rice yields in the CRU treatments and in the U treatment were similar in our study (Fig. 2). However, Zheng *et al*.^44^ reported that short-term CRU treatments led to greater crop yields, and thus increased the incorporation of residues into the soil compared to the U treatment. Mi *et al*.^46^ found that 3-year application of CRU had similar soil AP and AK concentrations with split application of urea. In our study, no significant differences were observed in soil AP and AK between the U and CRU treatments.

**Table 2:** General description of the geography and soil properties (0–20 cm) at the two experimental sites.

	Lincheng	Xintang
Location	30°56ʹN, 119°46′E	31°01′N, 119°54′E
Mean annual precipitation (mm)	1328	1300
Mean annual temperature (°C)	16.4	17.5
Soil classification	Anthrosols	Anthrosols
SOM (g kg^−1^)	29.0	38.1
TN (g kg^−1^)	1.91	2.31
pH (soil: water = 1: 2.5)	5.99	5.69
AHN (mg kg^−1^)	188.7	255.9
AP (mg kg^−1^)	7.2	27.3
AK (mg kg^−1^)	49.8	166.3

SOM, TN, AHN, AP and AK represent soil organic matter, total nitrogen, alkaline hydrolyzable nitrogen, available phosphorus and available potassium, respectively.

**Table 3:** The N concentration in and N uptake of rice were affected by the different treatments at the two experimental sites during the 2015 to 2017 rice growing seasons.

Year	Treatment	Lincheng					Xintang				
		N concentration (mg kg^−1^)		N uptake (kg ha^−1^)			N concentration (mg kg^−1^)		N uptake (kg ha^−1^)		
		grain	straw	grain	straw	total	grain	straw	grain	straw	total
2015	CK	9.7 ± 3.4 a	5.2 ± 0.7 b	65.1 ± 24.2 b	31.8 ± 4.7 b	97.0 ± 28.2 b	8.8 ± 0.4 b	5.5 ± 0.6 b	52.9 ± 3.8 b	30.0 ± 4.3 b	82.9 ± 8.0 b
	U	11.3 ± 1.6 a	8.5 ± 1.7 a	92.2 ± 12.2 a	63.2 ± 13.1 a	155.4 ± 13.3 a	9.5 ± 0.7 ab	8.2 ± 1.1 a	67.1 ± 9.8 a	52.8 ± 11.5 a	119.9 ± 20.5 a
	RCU	10.2 ± 2.0 a	8.7 ± 1.4 a	84.7 ± 15.8 a	65.2 ± 9.1 a	149.9 ± 21.6 a	9.9 ± 0.3 a	7.9 ± 2.1 a	74.2 ± 2.4 a	53.8 ± 14.4 a	128.0 ± 13.7 a
	PCU	10.8 ± 22 a	7.5 ± 0.5 a	88.7 ± 17.1 a	55.8 ± 5.2 a	144.5 ± 20.3 a	10.1 ± 0.7 a	8.1 ± 1.4 a	76.1 ± 7.2 a	54.7 ± 7.4 a	130.8 ± 7.9 a
2016	CK	7.0 ± 0.5 b	4.7 ± 0.6 b	46.3 ± 3.0 b	28.7 ± 4.5 c	75.1 ± 4.5 b	13.6 ± 2.7 b	6.7 ± 1.1 b	76.0 ± 13.7 b	23.7 ± 6.0 b	99.6 ± 19.7 b
	U	10.3 ± 1.4 a	6.6 ± 0.5 a	83.4 ± 13.0 a	48.2 ± 4.1 b	131.6 ± 15.8 a	15.7 ± 1.5 a	9.5 ± 0.8 a	113.2 ± 8.0 a	47.4 ± 5.2 a	160.6 ± 11.3 a
	RCU	9.6 ± 1.5 a	7.9 ± 1.3 a	80.6 ± 11.7 a	60.2 ± 9.7 a	140.8 ± 19.9 a	15.6 ± 0.7 a	8.6 ± 0.2 a	115.3 ± 15.0 a	43.0 ± 7.3 a	158.2 ± 19.9 a
	PCU	10.5 ± 1.4 a	6.8 ± 0.4 a	86.1 ± 10.5 a	50.6 ± 1.7 ab	136.7 ± 10.1 a	14.3 ± 0.9 ab	8.5 ± 0.4 a	108.7 ± 12.1 a	37.0 ± 3.7 ab	145.7 ± 12.2 a
2017	CK	10.8 ± 0.9 b	5.1 ± 1.2 b	78.1 ± 6.8 b	33.3 ± 7.6 c	111.3 ± 11.4 b	11.9 ± 0.3 a	6.9 ± 1.6 b	77.7 ± 5.3 b	40.9 ± 10.6 b	118.6 ± 14.7 b
	U	12.6 ± 2.5 a	8.1 ± 0.8 a	110.1 ± 21.0 a	64.4 ± 5.9 a	174.6 ± 24.9 a	13.3 ± 0.6 a	8.4 ± 1.1 a	109.2 ± 11.3 a	62.7 ± 6.6 a	171.9 ± 13.7 a
	RCU	12.4 ± 1.0 a	6.4 ± 0.7 ab	110.3 ± 11.0 a	51.6 ± 5.8 ab	162.0 ± 8.8 a	13.2 ± 1.9 a	7.3 ± 1.0 ab	107.1 ± 17.1 a	54.1 ± 6.4 ab	161.2 ± 12.6 a
	PCU	12.7 ± 0.7 a	5.9 ± 1.2 b	109.1 ± 3.7 a	46.1 ± 8.5 b	155.2 ± 11.0 a	12.3 ± 1.6 a	6.9 ± 0.9 b	104.8 ± 17.9 a	51.1 ± 6.3 ab	155.9 ± 22.1 a

The values are presented as the mean ± standard deviation (n = 3). Different letters in the same columns at the same site represent significant differences at the level of P < 0.05. CK, U, RCU and PCU represent no N application, conventional urea, resin-coated urea and polyurethane-coated urea, respectively.

**Table 4:** Indices of N use efficiency were affected by the different treatments at the two experimental sites during the 2015 to 2017 rice growing seasons.

Site	Treatment	2015			2016			2017		
		NARE (%)	NAE (kg kg^−1^)	NPFP (kg kg^−1^)	NARE (%)	NAE (kg kg^−1^)	NPFP (kg kg^−1^)	NARE (%)	NAE (kg kg^−1^)	NPFP (kg kg^−1^)
Lincheng	U	21.6 ± 4.9 a	5.3 ± 0.2 a	30.1 ± 0.2 a	20.9 ± 5.9 a	5.2 ± 0.5 b	29.9 ± 0.5 b	23.4 ± 9.2 a	5.7 ± 0.3 a	32.3 ± 0.3 b
	RCU	24.5 ± 10.0 a	7.2 ± 0.9 a	38.3 ± 0.9 a	30.4 ± 9.2 a	8.0 ± 0.4 a	38.8 ± 0.4 a	23.5 ± 4.1 a	7.7 ± 1.4 a	41.1 ± 1.4 a
	PCU	22.0 ± 9.4 a	7.0 ± 1.8 a	38.1 ± 1.8 a	28.5 ± 4.7 a	7.2 ± 1.2 a	38.1 ± 1.2 a	20.3 ± 5.1 a	6.5 ± 1.0 a	39.9 ± 1.0 a
Xintang	U	13.7 ± 7.6 b	3.7 ± 1.3 b	26.0 ± 2.3 b	22.6 ± 4.2 a	5.9 ± 1.2 b	26.7 ± 1.2 b	19.8 ± 5.1 a	6.4 ± 1.9 b	30.5 ± 1.9 b
	RCU	20.9 ± 6.3 a	6.8 ± 1.7 a	34.7 ± 1.7 a	27.1 ± 9.2 a	8.2 ± 3.4 ab	34.2 ± 3.4 a	19.7 ± 5.8 a	7.5 ± 0.7 ab	37.6 ± 0.7 a
	PCU	22.2 ± 3.7 a	6.8 ± 1.5 a	34.7 ± 1.5 a	21.3 ± 5.7 a	9.3 ± 1.7 a	35.2 ± 1.7 a	17.3 ± 1.2 a	9.4 ± 2.3 a	39.4 ± 2.3 a

The values are presented as the mean ± standard deviation (n = 3). Different letters in the same columns at the same site represent significant differences at the level of P < 0.05. U, RCU and PCU represent conventional urea, resin-coated urea and polyurethane-coated urea, respectively. NARE, NAE and NPFP represent N apparent recovery efficiency, N agronomic efficiency and N partial factor productivity, respectively.

**Table 5:** Yield components of rice were affected by different treatments at the two experimental sites during the 2015 to 2017 rice seasons.

Year	Treatment	Lincheng				Xintang			
		Panicles per m^2^	Grains per panicle	Grain filling (%)	1000-grain weight (g)	Panicles per m^2^	Grains per panicle	Grain filling (%)	1000-grain weight (g)
2015	CK	258.0 ± 13.7 b	128.7 ± 25.7 b	87.6 ± 6.3 a	25.9 ± 0.1 a	209.8 ± 10.6 b	131.9 ± 6.5 ab	96.0 ± 3.0 a	25.2 ± 0.1 a
	U	294.0 ± 18.7 ab	144.3 ± 4.2 a	83.0 ± 2.3 a	25.5 ± 0.2 b	235.3 ± 8.0 a	146.6 ± 8.8 a	98.4 ± 0.6 a	25.1 ± 0.1 a
	RCU	294.0 ± 10.4 ab	150.7 ± 5.5 a	86.8 ± 2.8 a	25.5 ± 0.3 b	256.7 ± 19.2 a	129.6 ± 4.6 b	97.9 ± 1.1 a	25.1 ± 0.1 a
	PCU	297.0 ± 32.4 a	142.3 ± 9.5 a	87.2 ± 4.6 a	25.5 ± 0.2 b	257.3 ± 12.5 a	138.4 ± 1.0 ab	94.5 ± 5.5 a	25.1 ± 0.1 a
2016	CK	328.5 ± 31.5 b	99.8 ± 13.5 a	93.0 ± 2.9 a	25.1 ± 0.2 a	185.4 ± 22.7 b	181.7 ± 6.7 a	71.9 ± 2.6 ab	25.3 ± 0.1 a
	U	378.0 ± 19.0 a	121.0 ± 12.2 a	93.4 ± 1.9 a	25.0 ± 0.1 a	269.9 ± 8.3 a	180.3 ± 11.2 a	68.2 ± 1.1 b	25.0 ± 0.1 a
	RCU	394.5 ± 49.4 a	100.1 ± 17.0 a	96.6 ± 0.8 a	25.0 ± 0.4 a	261.8 ± 14.1 a	174.3 ± 10.1 a	70.6 ± 0.8 ab	25.0 ± 0.0 a
	PCU	390.9 ± 53.8 a	111.8 ± 15.1 a	94.2 ± 2.5 a	25.2 ± 0.2 a	231.3 ± 33.9 a	187.3 ± 8.5 a	74.1 ± 3.3 a	25.1 ± 0.1 a
2017	CK	432.0 ± 18.3 b	71.3 ± 1.5 c	86.5 ± 0.7 a	23.9 ± 0.4 a	260.7 ± 32.2 a	106.0 ± 4.6 b	78.7 ± 1.3 ab	25.1 ± 0.3 a
	U	513.6 ± 18.7 a	83.0 ± 1.7 ab	76.1 ± 1.4 b	23.4 ± 0.3 a	275.4 ± 8.2 a	125.7 ± 1.2 a	80.5 ± 1.5 a	24.6 ± 0.3 a
	RCU	498.4 ± 26.9 a	86.7 ± 3.1 a	81.8 ± 4.6 a	24.2 ± 0.2 a	312.7 ± 26.6 a	115.7 ± 4.0 a	73.9 ± 1.8 c	24.5 ± 0.2 a
	PCU	509.1 ± 29.4 a	77.7 ± 6.0 bc	83.8 ± 2.2 a	23.6 ± 0.2 a	297.2 ± 41.1 a	121.7 ± 8.1 a	76.3 ± 2.0 bc	24.8 ± 0.5 a

The values are presented as the mean ± standard deviation (n = 3). Different letters in the same columns at the same site represent significant differences at the level of P < 0.05. CK, U, RCU and PCU represent no N application, conventional urea, resin-coated urea and polyurethane-coated urea, respectively.

**Table 6:** Soil pH, SOM, TN, AHN, AP and AK at the 0–20 cm depth were affected by different treatments at the two experimental sites in 2016.

Site	Treatment	pH	SOM (g kg^−1^)	TN (g kg^−1^)	AHN (mg kg^−1^)	AP (mg kg^−1^)	AK (mg kg^−1^)
Lincheng	CK	5.61 ± 0.11 a	33.76 ± 2.63 a	2.06 ± 0.11 a	217.45 ± 13.58 a	7.75 ± 2.06 a	55.33 ± 13.58 a
	U	5.60 ± 0.11 a	31.36 ± 1.78 a	1.96 ± 0.08 a	201.68 ± 8.04 a	6.65 ± 0.59 a	37.33 ± 14.64 a
	RCU	5.48 ± 0.30 a	32.89 ± 1.13 a	2.03 ± 0.07 a	204.40 ± 15.07 a	6.22 ± 1.21 a	40.67 ± 7.23 a
	PCU	5.35 ± 0.28 a	33.66 ± 2.34 a	2.08 ± 0.15 a	213.10 ± 19.11 a	7.08 ± 1.48 a	46.33 ± 14.57 a
Xintang	CK	5.28 ± 0.16 a	43.54 ± 1.37 a	2.54 ± 0.05 a	239.22 ± 37.60 a	16.27 ± 2.37 a	108.67 ± 6.11 a
	U	5.03 ± 0.29 ab	41.42 ± 2.93 a	2.42 ± 0.13 ab	201.56 ± 12.36 a	15.02 ± 4.34 a	99.33 ± 12.42 a
	RCU	4.86 ± 0.13 b	38.20 ± 4.46 a	2.23 ± 0.21 b	195.72 ± 26.58 a	14.24 ± 4.63 a	94.00 ± 18.19 a
	PCU	4.85 ± 0.14 b	41.04 ± 2.97 a	2.35 ± 0.11 b	212.70 ± 34.29 a	17.64 ± 2.25 a	99.33 ± 11.59 a

The values are presented as the mean ± standard deviation (n = 3). Different letters in the same columns at the same site represent significant differences at the level of P < 0.05. CK, U, RCU and PCU represent no N application, conventional urea, resin-coated urea and polyurethane-coated urea, respectively. SOM, TN, AHN, AP and AK represent soil organic matter, total N, alkaline hydrolyzable N, available P and available K, respectively.

## Conclusion

The results of present study showed that reductions in the current application rates of N fertilizer by 20% and the use of single basal applications of CRU (RCU and PCU) could maintain rice grain yields at levels that is similar to those resulting from split applications of urea in the middle and lower Yangtze River basin. In addition, in most cases, there were no significant differences in the N concentration in the grain among the RCU, PCU and U treatments. The yield component analysis revealed that, compared to those in the CK treatment, the higher rice grain yields from the N fertilizer treatments were attributed mainly to an increased number of panicles per m^2^ in response to the fertilizer treatments. The NARE, NAE and NPFP mostly increased in the CRU treatments compared to that in the U treatment at both sites. Future work should focus on studying the application rates of CRU and their effect on rice grain yields and N loss in the paddy field.

## Materials and Methods

### Study site

Field experiments were conducted in Lincheng town (LC) and Xintang town (XT) of Changxing City, Zhejiang Province, southeast China, from 2015 to 2017. These regions have a subtropical humid monsoon climate. The information on the climate and soil properties (0–20 cm) of the two sites is detailed in Table 2. The dominant cropping system was a rice–oilseed rape rotation at both sites. The mean temperature and rainfall data during the rice growing season are shown in Fig. 1.

### Experimental design

The field experiments were established as a completely randomized block design, with three replicates. The plots were 30 m^2^ (5 × 6 m) in size, and the ridges (0.3 m wide and 0.2 m high) were covered with plastic film at all sites. In each plot, an individual inlet and outlet were set up for irrigation and drainage. The four treatments were as follows: (1) CK (no N fertilizer application), (2) U (conventional urea), (3) RCU (resin-coated urea), and (4) PCU (polyurethane-coated urea). The application rate and timing of the various fertilizers are shown in Table 1. For the U treatment, 50% of the N fertilizer was broadcast by hand at one day before transplanting and then was incorporated into the soil (0–15 cm depth); another 20% was broadcast at the tillering stage; and the remaining 30% was broadcast at the booting stage. For the CRU treatments (RCU and PCU), the N fertilizer was applied once as basal fertilizer by hand at one day before rice transplantation and then incorporated into the soil (0–15 cm depth). Superphosphate (containing 12% P_2_O_5_, 7% S and 12% Ca) and potassium chloride (containing 60% K_2_O) were used in this study as the P and K fertilizers, respectively. All the P and K fertilizers in all treatments were applied as basal fertilizers at one day before transplanting at both sites. The RCU (resin-coated urea containing 45% N) and PCU (polyurethane-coated urea containing 44% N) were provided by Shandong Kingenta Ecological Engineering Co. Ltd. and China Agricultural University, respectively. The theoretical release longevity of RCU and PCU was 90 and 60 days at 25 °C in water, respectively. The rice (*Oryza sativa* L.) varieties used in this study were No. *Xiushui* 33# in LC and No. *Jia* 67# in XT, both of which exhibited good adaptability and productivity in the region. Rice seedlings (30 days of age) were transplanted at a spacing of 30 cm × 20 cm at both sites. The water was irrigated in each plot 7 days prior to rice transplantation and was maintained at 5–7 cm until midseason aeration. After the end of aeration, flooding water was continually maintained at 5–7 cm and drained at 7 days before rice harvest. Pesticides and herbicides were applied according the practices of local farmers if necessary.

### Sampling and measurements

At maturity, all the rice was harvested by hand in each plot and divided into grain and straw by a threshing machine (Rongchang Machinery Manufacture Co., Ltd, Zhengzhou, China). The grain and straw samples were dried at 75 °C to a constant weight for the determination of the dry weights of the rice grain and straw. Twelve rice plants were collected randomly to assess the panicles and grains numbers per panicle. The grain filling percentage and 1000-grain weight were also determined. The dry plant samples were ground into powder using a grinder (Taisite Instrument Inc., Tianjin, China) and then passed through a 0.15-mm screen to determine the N concentration in the plant. For the N concentration analysis, grain and straw samples were digested with H_2_SO_4_-H_2_O_2_ at 270 °C and then were measured using the Kjeldahl method^47^.

After the rice harvest in 2016, five soil cores (0–20 cm depth) were randomly collected in each plot and mixed into a composite sample. The fresh soil samples were sieved to pass through a 2-mm screen and then air-dried to determine the soil chemical properties. The soil pH was measured using a pH meter (soil: water = 1: 2.5), and the SOM was measured by oxidation with both potassium dichromate and concentrated sulfuric acid and titration with ferrous ammonium sulfate^47^. The total N content (TN) was determined according to the Kjeldahl digestion method^47^. The AHN content was determined according to Roberts *et al*.^48^. The soil available P (AP) was measured using the Olsen-P method, which is based on the extraction of soil with 0.5 M sodium bicarbonate^49^ and the available K (AK) was extracted by 1 M ammonium acetate and measured by a flame photometer.

### N use efficiencies

The parameters of NUE used in this study included the following: N apparent recovery efficiency (NARE, %), N agronomic efficiency (NAE, kg kg^−1^) and N partial factor productivity (NPFP, kg kg^−1^)^37,41,50^.

$$\begin{array}{ccc}{\rm{NARE}}\,( \% ) & = & \frac{{\rm{UN}}-{\rm{UCK}}}{{\rm{FN}}}\times 100 \% \\ {\rm{NAE}}\,({\rm{kg}}\,{{\rm{kg}}}^{-1}) & = & \frac{{\rm{YN}}-{\rm{YCK}}}{{\rm{FN}}}\\ {\rm{NPFP}}\,({\rm{kg}}\,{{\rm{kg}}}^{-1}) & = & \frac{{\rm{YN}}}{{\rm{FN}}}\end{array}$$

where U_N_ and U_CK_ are the total N uptake in the fertilized treatments and no N application treatment, respectively. F_N_ is the N fertilizer application rate. Y_N_ and Y_Ck_ are the rice grain yield in the fertilizer treatments and no N application treatment, respectively.

### Statistical analysis

Statistical analysis was performed using SPSS 16.0 (SPSS Inc., Chicago, USA) for analysis of variance (ANOVA). A two-way ANOVA was used to evaluate the effects of site, year, treatment and their interaction on the yield, N concentration, N uptake and NUE of rice. The data were compared among the four treatments by one-way ANOVA in conjunction with the least significant difference (LSD) test at *p* < 0.05.

## Supplementary information

Supplementary Material.
