# Supplementary material for: Impact of controlled-release urea on rice yield, nitrogen use efficiency and soil fertility in a single rice cropping system
Source: Sci Rep. 2020 Jun 26;10:10432. doi: 10.1038/s41598-020-67110-6 (PMC7320022; doi:10.1038/s41598-020-67110-6)
Supplement: Supplementary file 1 — Supplementary Material. [file 41598_2020_67110_MOESM1_ESM.docx]

**Impact of controlled-release urea on rice yield, nitrogen use efficiency and soil fertility in a single rice cropping system**

Zhaoming Chen^1^, Qiang Wang^1,^ *, Junwei Ma^1^, Ping Zou^1^ & Lina Jiang^1^

^1^ Institute of Environmental Resources and Soil Fertilizer, Zhejiang Academy of Agricultural Sciences, Hangzhou, 310021, China.

* Corresponding author: Qiang Wang

E-mail: [qwang0571@126.com](mailto:qwang0571@126.com) (Q.W.)

Tel: +86 0571 86404385

**Table S1**. Results from a two-way ANOVA on the effects of year, treatment and their interaction on the crop biomass, N concentration, N uptake, yield component and N use efficiency at the two experiment sites during the 2015 to 2017 rice seasons.

| Site | Lincheng | | | Xintang | | |
| --- | --- | --- | --- | --- | --- | --- |
| Source of variance | Year  (Y) | Treatment  (T) | Y × T | Year  (Y) | Treatment (T) | Y × T |
| df | 1 | 3 | 3 | 1 | 3 | 3 |
| Grain yield | NS | *** | NS | NS | *** | NS |
| Aboveground biomass | NS | *** | NS | *** | *** | NS |
| Grain N concentration | NS | NS | NS | *** | NS | NS |
| Straw N concentration | * | *** | NS | NS | ** | NS |
| N uptake by grain | NS | ** | NS | *** | *** | NS |
| N uptake by straw | * | *** | NS | ** | ** | NS |
| Total N uptake | * | *** | NS | ** | *** | NS |
| NARE | NS | NS | NS | NS | NS | NS |
| NAE | NS | ** | NS | NS | * | NS |
| NPFP | NS | *** | NS | NS | *** | NS |
| Panicles per m^2^ | *** | * | NS | NS | *** | * |
| Grains per panicle | *** | NS | NS | *** | NS | NS |
| Grain filling percentage | *** | NS | NS | *** | NS | * |
| 1000-grain weight | *** | NS | NS | NS | ** | NS |

NARE, NAE and NPFP represent N apparent recovery efficiency, N agronomic efficiency and N partial factor productivity, respectively. NS Not significant; * Significant at *p* < 0.05; ** Significant at *p* < 0.01; *** Significant at *p* < 0.001.
